# MFSynDCP: multi-source feature collaborative interactive learning for drug combination synergy prediction

**DOI:** 10.1186/s12859-024-05765-y

**Published:** 2024-04-01

**Authors:** Yunyun Dong, Yunqing Chang, Yuxiang Wang, Qixuan Han, Xiaoyuan Wen, Ziting Yang, Yan Zhang, Yan Qiang, Kun Wu, Xiaole Fan, Xiaoqiang Ren

**Affiliations:** 1 School of Software, Taiyuan University of Technology, Taiyuan, Shanxi China; 2 College of Computer Science and Technology (College of Data Science), Taiyuan University of Technology, Taiyuan, Shanxi China; 3 School of Computing, University of Leeds, Leeds, West Yorkshire UK; 4 Information Management Department, Shanxi Provincial People’s Hospital, Taiyuan, Shanxi China

**Keywords:** Drug combination, Synergistic effect, Graph attention network, Anti-tumor, Deep learning

## Abstract

Drug combination therapy is generally more effective than monotherapy in the field of cancer treatment. However, screening for effective synergistic combinations from a wide range of drug combinations is particularly important given the increase in the number of available drug classes and potential drug-drug interactions. Existing methods for predicting the synergistic effects of drug combinations primarily focus on extracting structural features of drug molecules and cell lines, but neglect the interaction mechanisms between cell lines and drug combinations. Consequently, there is a deficiency in comprehensive understanding of the synergistic effects of drug combinations. To address this issue, we propose a drug combination synergy prediction model based on multi-source feature interaction learning, named MFSynDCP, aiming to predict the synergistic effects of anti-tumor drug combinations. This model includes a graph aggregation module with an adaptive attention mechanism for learning drug interactions and a multi-source feature interaction learning controller for managing information transfer between different data sources, accommodating both drug and cell line features. Comparative studies with benchmark datasets demonstrate MFSynDCP's superiority over existing methods. Additionally, its adaptive attention mechanism graph aggregation module identifies drug chemical substructures crucial to the synergy mechanism. Overall, MFSynDCP is a robust tool for predicting synergistic drug combinations. The source code is available from GitHub at https://github.com/kkioplkg/MFSynDCP.

## Introduction

In recent years, the field of malignant tumor biology has yielded a multitude of effective anticancer drugs. However, the inherent heterogeneity of tumors and the development of drug resistance often render single-drug therapies targeting individual markers ineffective [1]. In contrast, drug combination therapies [2–4] have shown great potential to improve efficacy. By acting on multiple targets and pathways, they can effectively improve efficacy, reduce side effects, and overcome drug resistance [5, 6] However, there is a risk of antagonistic interactions or severe adverse reactions with some drug combinations, posing potential threats to patient health. Oncology, as one of the largest disease areas for global drug development, it has become particularly important to predict effective synergistic drug combinations from the huge number of anti-tumor drugs.

Traditional methods of predicting drug combinations primarily rely on numerous time-consuming and expensive clinical trials [7], which may cause patients to receive some unnecessary treatments and cause psychological or physiological harm. With the development of high-throughput drug screening technologies [8–10], researchers have accelerated the search for drug combinations with synergistic effects by using automated testing platforms and large-scale compound libraries to conduct extensive drug combination screening across hundreds of cancer cell lines. However, high-throughput drug screening methods are mainly based on in vitro cell models or animal models, and they ignore the complex interaction networks between drugs, biomolecules, and signaling pathways. These methods are unable to fully simulate the complexity of drug interactions in the human body [11]. Additionally, it is impractical to screen all possible drug combinations using this approach [12].

Recently, the advancement of artificial intelligence [13] and the availability of large-scale datasets have made it feasible to explore machine learning models [14–16] or deep neural networks [17] for drug combination predictions, which can reduce the cost of drug experiments while improving the prediction of synergistic effects of drug combinations. Mei [18] proposed an independent machine learning framework to simultaneously predict synergistic, antagonistic, and additive effects of drugs. This framework represents drug pairs through simple graphs of drug-targeted genes and cellular processes, thereby effectively explaining the molecular mechanisms behind drug interactions and reducing data complexity. Janizek et al. [19] utilized feature attribution methods, improving the quality of explanations by using a collection of interpretable machine learning models, and discovered hematopoietic differentiation characteristic drug combinations with therapeutic synergistic effects. Julkunen et al. [20] proposed a machine learning framework, comboFM, for predicting responses to drug combinations in preclinical studies. It models high-order tensor drug interactions specific to cellular contexts and uses powerful factorization machines to efficiently learn the latent factors of the tensor, predicting responses to new combinations in cells not yet tested. However, training a good machine model for the prediction of drug combination synergy tasks requires specialized domain knowledge and experience to manually select and construct relevant features [21].

The rapid development of deep learning has provided new possibilities for addressing these challenges. Deep learning models can automatically learn high-level abstract features from raw data [22], eliminating the reliance on manually designed features and better capturing complex relationships and nonlinear patterns in data. Various neural network models have been employed in the field of drug combination prediction. Preuer et al. [23] introduced a deep learning model named DeepSynergy, which uses chemical and genomic information as input and employs a normalization strategy to account for the heterogeneity of the input data. This was the first attempt to utilize deep learning in this field, and the performance of this model surpassed traditional machine learning methods. Rafiei et al. [24] utilized multimodal deep learning and transformer for multitask predictions, including drug-target interactions, toxic effects, and synergistic effects of drug combinations. Yang et al. [25] proposed a deep learning model called GraphSynergy, by adopting space-based graph convolutional network components and attention mechanisms, encodes high-order topological relationships in the Protein–Protein Interaction (PPI) network of protein modules. The approach focuses on identifying crucial proteins involved in biomolecular interactions within the PPI network, as well as interactions between drug combinations and cancer cell lines, with the aim of predicting synergistic drug combinations. However, these methods primarily focus on separately extracting features from drug molecules and cell line structures, without adequately considering the integration of cell line-drug combination pairs. This leads to a limitation in the model's ability to learn associated patterns from the data. Additionally, these methods have certain limitations in focusing on important substructures within drug molecules, which may pose challenges to the biological interpretation of the predictions.

Based on the considerations mentioned above, we propose a model for predicting the synergy of drug combinations based on multi-source feature interactive learning, named MFSynDCP. Specifically, we introduce a deep graph attention neural network to automatically learn and extract high-dimensional features of drugs. Then, we propose an adaptive attention mechanism graph aggregation module to capture the drug substructures that are most critical for predicting the synergistic effects of drug combinations. Additionally, we introduce a multi-source feature interactive learning controller, which incorporates a parameter self-learning gating structure within the controller to regulate information transfer between different data sources, thereby flexibly handling diverse features. Finally, we compared our method with recent deep learning prediction models, and the results show that our approach, MFSynDCP, has significant advantages in predicting drug combination synergy compared to recent deep learning models. Specifically, our model exhibits superior accuracy, enhanced predictive capabilities, and increased stability.

## Materials and methods

### Dataset

We used the large-scale tumor screening drug combination dataset published by O'Neil et al. [26] in 2016 as our benchmark dataset. This dataset involves screening of 583 different combinations of 22 experimental drugs and 16 approved drugs across 39 cancer cell lines, comprising 23,052 triplets, each consisting of two drugs and a cancer cell line. The Loewe Additivity [27, 28] scores for each pair of drugs were calculated using the Combenefit tool based on the 4 × 4 dose–response matrix in the dataset. The effect of each individual drug at the same dose served as a baseline, and the scores for the synergistic or antagonistic effects of drug combinations were quantitatively calculated by comparing the effect of the drug combination with the expected additive effect. According to the Loewe scores, combinations with scores greater than zero were considered synergistic, while those with scores less than zero were considered antagonistic. Considering the presence of noise, which results in synergy scores close to zero, we adopted a more stringent threshold for finer classification of these combinations. We chose 10 as the threshold to classify the drug pair-cell line triplets, considering drug combinations with Loewe scores above 10 as synergistic and those with scores below zero as antagonistic. Ultimately, a balanced benchmark dataset was obtained, comprising 12,415 unique drug pair-cell line combinations, covering 36 anti-cancer drugs and 31 human cancer cell lines.

In this study, the SMILES (Simplified Molecular Input Line Entry System) sequence data of drugs [29] were sourced from the DrugBank database [30]. We obtained the SMILES expressions of the required drugs from the DrugBank database and used RDKit [31] to convert the SMILES sequences of the drugs into corresponding molecular graph representations. Drug compounds are viewed as graphical structures based on interactions between atoms. The transformed molecular graphs depict the overall structure of the molecules through a series of atoms and bonds, illustrating the connections and spatial arrangement of atoms. In these graphs, vertices represent the atoms in the drug structure, and edges indicate the chemical bonds connecting these atoms.

In this paper, relevant gene expression data for cancer cell lines were obtained from the Cancer Cell Line Encyclopedia (CCLE) [32]. Considering factors such as gene length and sequencing depth, the gene expression data were standardized using Transcripts Per Million (TPM) [33] to normalize expression levels. This normalization process ensures more accurate and reliable comparisons between different genes and cell lines. By standardizing gene expression data in this manner, it becomes feasible to conduct in-depth analyses across various cell lines, facilitating a better understanding of the biological characteristics and behaviors of different cancer types at the genetic level.

### MFSynDCP

**Fig. 1:**
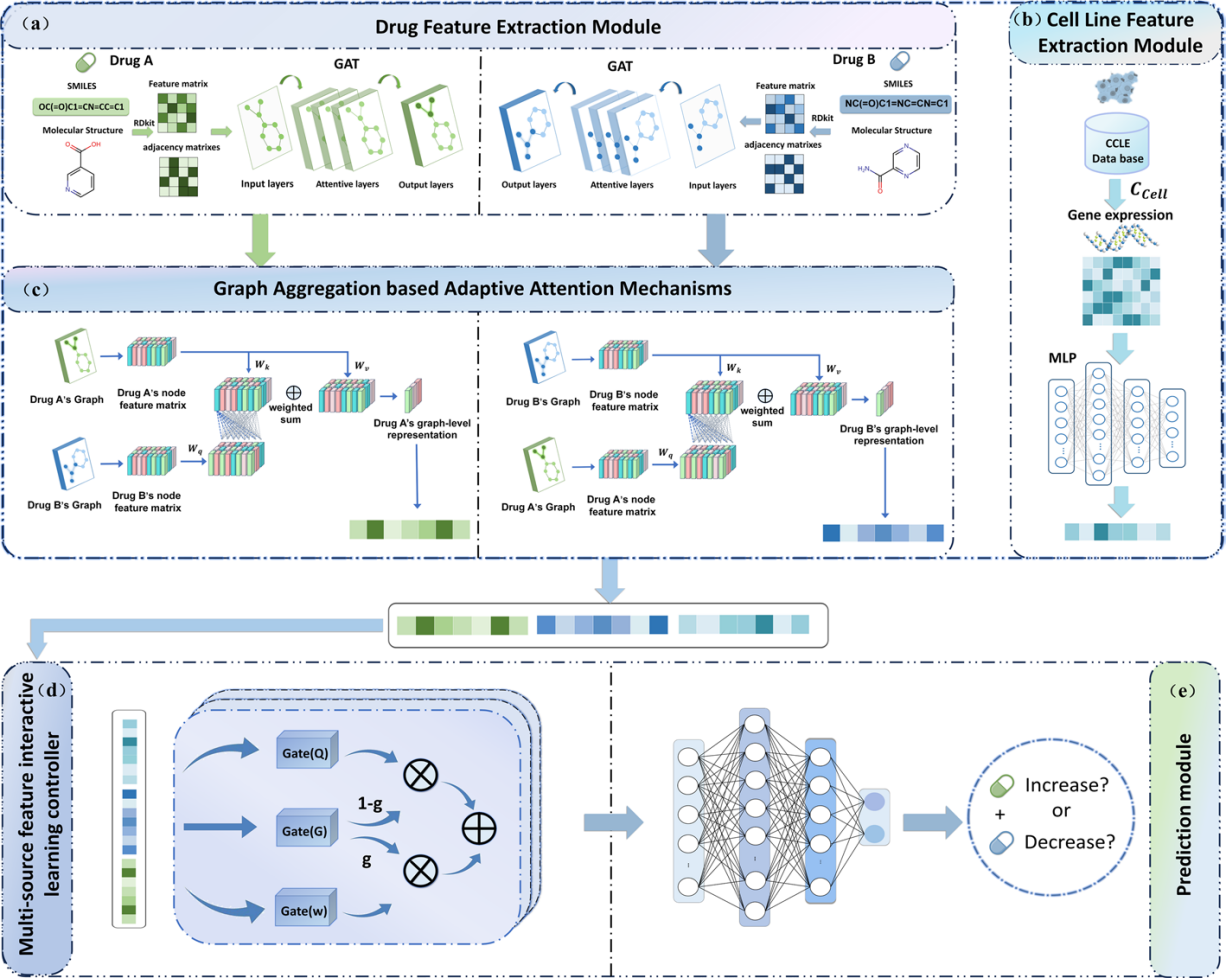
The workflows of the MFSynDCP model framework process. Drug molecular graphs are generated based on the SMILES sequences of drugs, and their feature embeddings are obtained using a GAT. Additionally, feature embeddings of cancer cell line gene expression profiles are acquired using a MLP. The embedding vectors of the drugs and cell lines are then concatenated and input into a multi-source feature interactive learning controller for the fusion of multi-source features. Finally, the fused features are fed into the prediction module for predicting the synergistic effects of drug combinations

Figure 1 illustrates the end-to-end learning framework MFSynDCP proposed for predicting the synergistic effects of drug combinations. The model comprises five parts: a feature extraction module for drugs (Fig. 1a), a feature extraction module for cell lines (Fig. 1b), a graph aggregation module based adaptive attention mechanism (Fig. 1c), a multi-source feature interactive learning controller (Fig. 1d), and a synergy prediction module (Fig. 1e). The process begins with the transformation of drug SMILES strings into molecular structure graphs using RDKit. The input layer receives the molecular structure graphs of two drugs, as well as the gene expression profiles of the cell lines affected by these drugs. A Graph Attention Network (GAT) extracts features from the nodes and edges of the drug molecular graphs. The design includes an adaptive attention mechanism graph aggregation module, which dynamically focuses on key information within the drug pair and comprehensively captures important interaction features between the drugs. A Multi-Layer Perceptron (MLP) encodes the genomic features of cancer cells, utilizing nonlinear transformations and mappings to capture and extract potential gene expression information. To fully consider the intrinsic correlation and interaction between them, their feature vectors are concatenated and processed through a multi-source feature interactive learning controller. This controller efficiently handles the concatenated feature vectors, delving into and conveying deeper-level features, ensuring the smooth integration of multi-source heterogeneous data. Finally, the processed integrated features are passed through a linear layer to output the predicted synergy scores of the drug pairs. Based on predetermined thresholds, the model determines whether the drug combination has a synergistic or antagonistic effect.

### Drug feature extraction based on GAT

We use the software RDkit to convert the drug’s SMILES string into a molecular graph, where nodes are atoms and edges are chemical bonds between atoms. The drug graph is defined as $G=(V,E)$, with $V$ being a set of $N$ nodes each represented by a d-dimensional vector, and E as a set of edges represented by the adjacency matrix A of the drug molecule's topological graph. ${x}_{i}\in V$ represents an atom, and ${e}_{ij}\in E$ represents a chemical bond between atoms. DeepChem [34], a cheminformatics software package that provides tools and algorithms for processing and analyzing chemical molecule data, is used to calculate atomic properties in each node of the drug molecular graph as initial features. Each atom ${x}_{i}$ is represented as a vector [${x}_{i1}$, ${x}_{i2}$, …${x}_{i5}$], where the elements of the vector correspond respectively to the atomic symbol, the number of adjacent atoms, the atom's implicit valence, the count of adjacent hydrogen atoms, and the atom's inclusion in a benzene ring structure.

When dealing with graph structures, traditional CNN models experience a significant decrease in performance in Euclidean space. In the task of predicting drug combinations, extracting feature representations of drugs from chemical molecular graphs is necessary, but traditional convolutional networks are not up to the task. Given that Graph Convolutional Networks (GCN), typically used for processing graph structures, treat each neighboring node as equally important and fail to capture the varying significance between nodes, we have adopted the GAT as the primary model for extracting drug molecular structure. By introducing graph attention layers in its architecture and utilizing a multi-head self-attention mechanism, the GAT learns advanced features of nodes in the graph. It dynamically allocates attention weights based on the relationships between nodes and their neighbors, thereby more accurately capturing the differences in importance between nodes and enhancing the representation capability of drug features.

For each vertex atom i in the transformed drug molecular graph, the correlation coefficient between each neighboring atom $j\in {N}_{i}$ and the atom i itself is calculated individually.

The process of calculating the correlation coefficient as follows:

1 $${{\text{e}}}_{{\text{ij}}}={\text{a}}(\left[{{\text{Wh}}}_{{\text{i}}}|\left|{{\text{Wh}}}_{{\text{j}}}\right]\right),{\text{j}}\in {{\text{N}}}_{{\text{i}}}$$

where $W\in {\mathbb{R}}^{{K}{\prime}\times K}$ represents a weight matrix, and the attention mechanism ${\text{a}}$ is a single-layer feedforward neural network, parameterized by the weight vector ${\overrightarrow{a}}^{T}\in {\mathbb{R}}^{2{K}{\prime}}$. The feature vector ${h}_{i}$ corresponds to the features of node ${\text{i}}$. The function $||$ concatenates the transformed features of atoms $i$ and $j$. Normalize the attention coefficients using the softmax function:

2 $${\mathrm{\alpha }}_{{\text{i}},{\text{j}}}={{\text{softmax}}}_{{\text{j}}}({{\text{e}}}_{{\text{ij}}})=\frac{{\text{exp}}({{\text{e}}}_{{\text{ij}}})}{{\sum }_{{\text{z}}\in {{\text{N}}}_{{\text{i}}}}{\text{exp}}({{\text{e}}}_{{\text{iz}}})}=\frac{{\text{exp}}({\text{LeakyReLU}}({\overrightarrow{{\text{a}}}}^{{\text{T}}}[{\text{W}}{\overrightarrow{{\text{h}}}}_{{\text{i}}}||{\text{W}}{\overrightarrow{{\text{h}}}}_{{\text{j}}}]))}{{\sum }_{{\text{z}}\in {\text{N}}}{\text{exp}}({\text{LeakyReLU}}({\overrightarrow{{\text{a}}}}^{{\text{T}}}[{\text{W}}{\overrightarrow{{\text{h}}}}_{{\text{i}}}||{\text{W}}{\overrightarrow{{\text{h}}}}_{{\text{j}}}]))}$$

Based on the calculated attention coefficients ${\alpha }_{i,j}$, the features are weighted and summed up, and then processed through the activation function ${\sigma }_{d}$. This process results in new features for each atom node $i$ after integrating the information from its neighboring atoms, using the multi-head attention mechanism, and the formula is as follows:

3 $${h}_{i}^{\mathrm{^{\prime}}}=|{|}_{m=1}^{M}{\sigma }_{d}\left({\sum }_{j\in Ni}{\alpha }_{ij}^{m}{W}^{m}{h}_{j}\right)$$

The proposed method for processing drug features allows for the simultaneous consideration of various aspects of feature information from neighboring atomic nodes. It dynamically allocates weights based on the relationships between nodes, focusing more on drug structures that play a key role in the synergistic interaction of drug-drug combinations in specific cell lines. This approach captures the interactions between different atomic nodes in the drug molecular graph, enhancing the representation capability of drug features. This improved representation is crucial during the feature fusion process, as it allows for a more accurate consideration of the contributions of different neighboring nodes in the drug molecular graph. Consequently, this enhances the accuracy and reliability of drug synergy prediction tasks.

### Adaptive attention mechanism graph aggregation module (AAGAM)

**Fig. 2:**
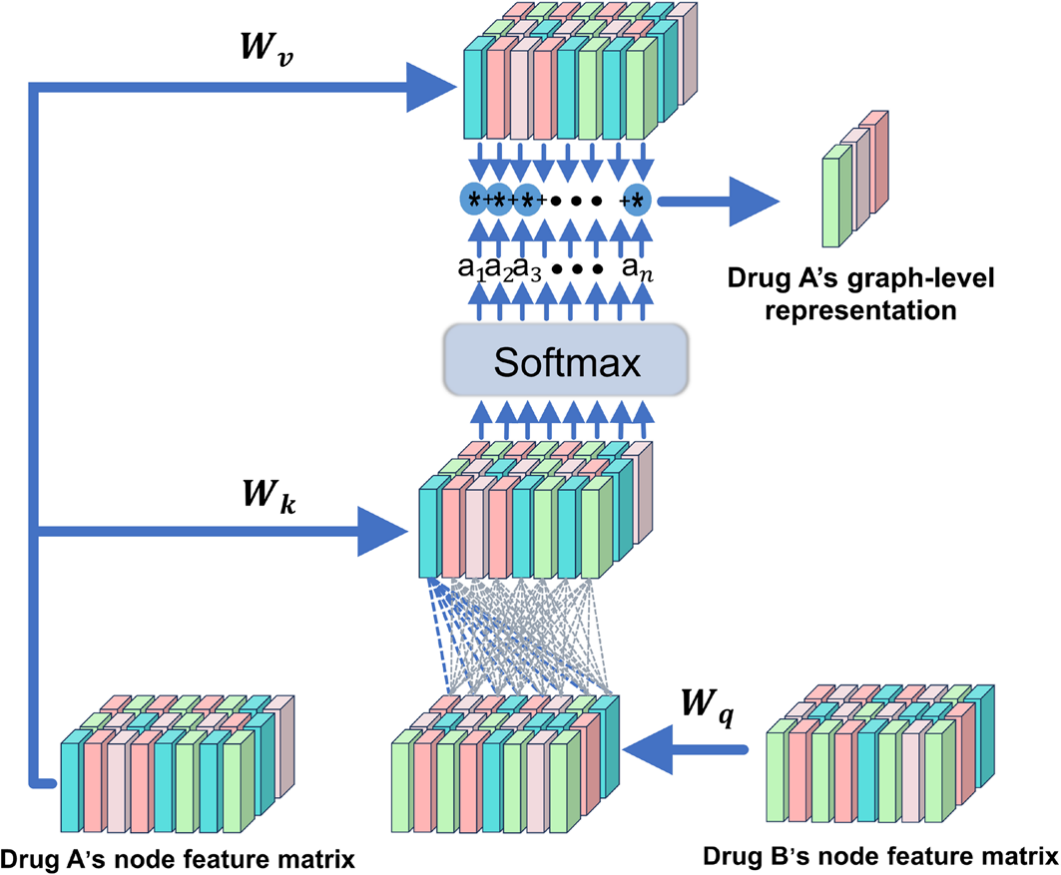
The calculation steps for the adaptive attention mechanism in graph aggregation. Taking drug A as an example, it involves a weighted summation of the embeddings of all nodes to compute the final graph-level representation of drug A

To better learn the interaction information between drug pairs and gain deeper insight into the impact mechanisms of drug structures on cancer cell genomes, we propose a graph aggregation module based on an adaptive attention mechanism. This module is designed to identify which drug substructures are more crucial for predicting the synergistic effects of drug pairs. By assigning attention scores to each substructure and performing a weighted summation of embedding vectors, the module reveals the molecular mechanisms underlying drug combinations' resistance or sensitivity responses in cancer treatment. These insights deepen our understanding of drug interactions and enable more accurate predictions of synergistic effects, we are able to not only extract interactive information between drug pairs but also identify the significant chemical substructures within drugs. As illustrated in Fig. 2, an attention score is assigned to each substructure of a drug. The module performs a weighted summation of the embedding vectors of all nodes, thereby obtaining an aggregated representation of the drug in graph form. This process allows for the extraction of interaction information between drug pairs and reveals the molecular mechanisms behind drug combinations' resistance or sensitivity responses in cancer treatment. The attention scores for the drug pairs are calculated using specific formulas as follows:

4 $${\mathrm{ S}}_{{\text{A}}}={\text{softmax}}\left({\sum }_{{\text{j}}=1}^{{\text{M}}}{\text{tanh}}({{\text{E}}}_{{\text{Ai}}}^{{\text{l}}}{{\text{W}}}_{{\text{k}}}({{\text{E}}}_{{\text{B}}}^{{\text{l}}}{{\text{W}}}_{{\text{q}}}{)}^{{\text{T}}}),{\text{j}}\right)$$

5 $${{\text{S}}}_{{\text{B}}}={\text{softmax}}\left({\sum }_{{\text{j}}=1}^{{\text{N}}}{\text{tanh}}({{\text{E}}}_{{\text{Bi}}}^{{\text{l}}}{{\text{W}}}_{{\text{k}}}({{\text{E}}}_{{\text{A}}}^{{\text{l}}}{{\text{W}}}_{{\text{q}}}{)}^{{\text{T}}}),{\text{j}}\right)$$

6 $${{\text{g}}}_{{\text{x}}}={\text{multiply}}\left({{\text{S}}}_{{\text{A}}},{{\text{E}}}_{{\text{Ai}}}{{\text{W}}}_{{\text{v}}}\right)$$

7 $${{\text{g}}}_{{\text{y}}}={\text{multiply}}\left({{\text{S}}}_{{\text{B}}},{{\text{E}}}_{{\text{Bi}}}{{\text{W}}}_{{\text{v}}}\right)$$

8 $${{\text{G}}}_{{\text{x}}}={\text{norm}}\left({\text{mean}}\left({{\text{E}}}_{{\text{Ai}}}\right)+{{\text{g}}}_{{\text{x}}}\right)$$

9 $${{\text{G}}}_{{\text{y}}}={\text{norm}}\left({\text{mean}}\left({{\text{E}}}_{{\text{Bi}}}\right)+{{\text{g}}}_{{\text{y}}}\right)$$

where ${E}_{Ai}^{l}$ and ${E}_{Bi}^{l}$ represent the graph embedding matrices of drugs A and B from the last layer of the GAT, respectively. M and N are the number of nodes in drugs A and B, respectively. ${W}_{k}$ and ${W}_{v}$ are the feature matrices learned by drug A through two linear layers, while ${W}_{q}$ is the feature matrix learned by drug B through a linear layer. Attention scores are used to assign weights to each node vector, with ${S}_{A}$ and ${S}_{B}$ being the calculated attention scores for drugs A and B, respectively. ${g}_{x}$ represents the weighted attention scores for ${W}_{v}$ for drug A. ${G}_{x}$ is the normalization of the sum of the average of ${E}_{Ai}^{l}$ along the first dimension and ${g}_{x}$, yielding the final graph-level representation. Similarly, ${G}_{y}$ is the final graph-level representation for drug B, obtained by normalizing the sum of the average of ${E}_{Bi}^{l}$ along the first dimension and ${g}_{y}$.

### Employing a MLP for cell line feature extraction

The CCLE gene expression profile we selected contains a wealth of gene information, making it challenging to construct a model that predicts synergy due to the high dimensionality of the feature space. To address the dimensional disparity between drug and cell line feature vectors, we turned to the “Landmark gene set” provided by the LINCS project [35]. Subsequently, our focus shifted towards identifying genes that overlapped between the landmark gene set and the CCLE gene expression profile for further exploration. Gene annotation information from the CCLE and the GENCODE [36] annotation database was utilized to remove redundant data and transcripts of non-coding RNA. In the end, a total of 954 genes were chosen from the initial expression profile to serve as input for the model.

For the collected gene expression features of cell line ${X}_{C}$, redundant gene data is removed using gene annotation information from the CCLE and GENCODE databases, ensuring the accuracy and reliability of the gene data. This results in a cell line feature matrix $C\in {\mathbb{R}}^{S\times U}$, where $S$ is the number of cell lines and $U$ is the dimension of features for each cell line. The latent features $v\in {\mathbb{R}}^{V}$ of cell line $C$ are captured through a ${q}_{c}$-layer fully connected neural network, and the formula is as follows:

10 $${\text{v}}={\text{tanh}}\left(\dots {\text{tanh}}\left({{\text{cW}}}_{{\text{c}}}^{0}\right)\dots {{\text{W}}}_{{\text{c}}}^{{{\text{q}}}_{{\text{c}}}}\right)$$

where ${W}_{c}^{{q}_{c}}$ represents the learnable weight parameters of the ${q}^{c}$ layer, $c$ is the gene expression data of cell line features, and $tanh$ is the activation function. After processing through the MLP, a new feature matrix ${C}^{\mathrm{^{\prime}}}$ is obtained, which is dimensionally consistent with the extracted drug features, facilitating their subsequent fusion.

### Multi-source feature interactive learning controller (MFIC)

Due to the involvement of multi-source data in the concatenated drug embedding vectors and cell line feature embedding vectors, including drug structure, biological activity, cell response, gene expression, etc., simply concatenating these vectors may not fully utilize the information from different data sources. Additionally, with the increase in network layers, the multitude of parameters can make the network difficult to train. To fully integrate and deeply mine the features of drugs and cell lines while accelerating network training, we propose a multi-source feature interactive learning controller. By introducing a gating structure into the network, it can control the flow of information between different data sources, flexibly handling various features. This approach better facilitates the fusion of multi-source data and ensures smooth data transmission across multiple layers.

**Fig. 3:**
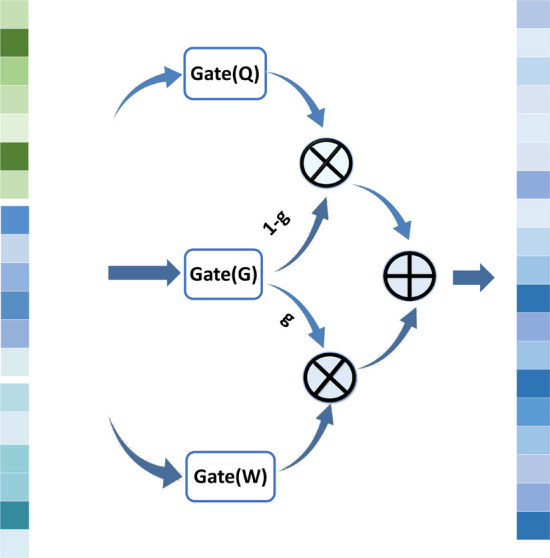
The Data Processing Process of MFIC

As shown in Fig. 3, the multi-source feature interactive learning controller divides the input data into two parts: one part undergoes nonlinear transformation, and the other part can pass through the layer without transformation. Based on this, the received information is selectively transmitted between layers, reducing the number of training parameters. For the vector F after concatenating drug features and cell line features, by setting the transformation gating$G\left(F\right)$, the model can effectively control two possible transformations, $W$ and$T$, on the input feature vector $F$ through self-learning. Specifically, $G\left(F\right)$ is set as a sigmoid transformation gate, converting its input values into probabilities between 0 and 1 to control the flow of input information. When the sigmoid gate output is close to 1, most of the input information undergoes transformation$T$; when the sigmoid gate output is close to 0, it indicates that most of the input information undergoes transformation$Q$. To simplify the model, the final output formula is as follows:

11 $${\text{y}}={\text{G}}\left({\text{F}}\right)\times {\text{W}}\left({\text{F}}\right)+\left(1-{\text{G}}\left({\text{F}}\right)\right)\times {\text{Q}}\left({\text{F}}\right)$$

where the transformation $W$ is set as a relu function, and the transformation $Q$ is set to perform a linear operation on the input. $y$ is the final output of the module, and the dimensions of $F$, $y$, $G$, and $Q$ are consistent. The addition of the sigmoid gating structure makes the form more flexible than the original, The simplified formula is as follows:

12 $$y = \left\{ {\begin{array}{*{20}l} {{\text{relu}}\left( {\text{F}} \right),} \hfill & {{\text{if}}\,{\text{sigmoid}}\left( {\text{F}} \right) = 1} \hfill \\ {{\text{linear}}\left( {\text{F}} \right),} \hfill & {{\text{if}}\,{\text{sigmoid}}\left( {\text{F}} \right) =0 } \hfill \\ \end{array} } \right.$$

The transformation gate $G$ automatically learns whether to use the relu function for transformation or to apply a linear operation in the current state. The Stochastic Gradient Descent (SGD) algorithm is used to adjust network parameters, as shown below:

13 $${W}_{t+1}={W}_{t}+\eta \frac{1}{N}{\sum }_{n=1}^{N}\theta \left(-{y}_{n}{W}_{t}{x}_{n}\right)\left({y}_{n}{x}_{n}\right)$$

where $W$ represents the weight parameters, $\eta$ is the learning rate, $N$ is the number of samples per training session, $\theta$ are the network parameters, and ${x}_{n}$ and ${y}_{n}$ are the input and output, respectively.

The gating mechanism, as an alternative to simple vector concatenation, more effectively utilizes information from multiple data sources such as drug structure, biological activity, cell response, and gene expression. It flexibly handles different features to achieve the fusion of multi-source data. Furthermore, by introducing a gating structure, it addresses the issue of increased parameter quantity making the network difficult to train, thereby accelerating network training and enhancing model performance.

### Predicting the synergistic effects between drug combinations and cell lines

In our model, we utilize a GAT and an adaptive attention mechanism to process drug embedding vectors, and a MLP to process cell line embedding vectors. These processed vectors are then concatenated to form a new vector. This concatenated vector is further refined through a multi-source feature interactive learning controller, ensuring smooth data transmission across multiple layers. The fused vector is then passed through an MLP and a softmax layer to generate a classification for the synergistic effects of the drug combination. This process is depicted in Fig. 1d.

During the training process of our model, ŷ represents the predicted synergistic score of the drug combination by the model, and y represents the actual synergistic score. We use cross-entropy as the loss function to measure the difference between predicted and actual values, and optimize the model's performance by minimizing this loss function. The specific loss function is as follows:

14 $${{\text{L}}}_{{\text{i}}1}=-\left[{{\text{y}}}_{{\text{i}}}{\text{log}}{\widehat{{\text{y}}}}_{{\text{i}}}+\left(1-{{\text{y}}}_{{\text{i}}}\right){\text{log}}\left(1-{\widehat{{\text{y}}}}_{{\text{i}}}\right)\right]$$

During the model training process, each sample is passed twice through the same network architecture, resulting in two different prediction outputs, ${\widehat{y}}_{1}^{i}$ and ${\widehat{y}}_{2}^{i}$. The adoption of dropout mechanism leads to the random elimination of some neurons during the network propagation. Consequently, ${\widehat{y}}_{1}^{i}$ and ${\widehat{y}}_{2}^{i}$ represent distinct prediction probabilities generated by the two different subnetworks formed by the network's two passes. This methodological approach of employing dual sub-networks introduces variability in the predictions, which substantially aids in enhancing the model’s generalization capacity and reducing the risk of overfitting.

To regularize the predictions from the two sub-networks, we minimize the Kullback–Leibler (KL) divergence between their respective output distributions. The KL divergence quantifies the difference between the probability distributions of ${\widehat{y}}_{1}^{i}$ and ${\widehat{y}}_{2}^{i}$, measuring how much one distribution deviates from the other. This regularization term encourages the two sub-networks to generate similar output distributions, promoting consistency and reducing uncertainty in the model's predictions, as shown below:

15 $${\text{KL}}({\widehat{{\text{y}}}}_{1}^{{\text{i}}}||{\widehat{{\text{y}}}}_{2}^{{\text{i}}})={\sum }_{{\text{i}}=1}^{{\text{N}}}[({\widehat{{\text{y}}}}_{1}^{{\text{i}}})({\text{log}}({\widehat{{\text{y}}}}_{1}^{{\text{i}}})-{\text{log}}({\widehat{{\text{y}}}}_{2}^{{\text{i}}}))]$$

Furthermore, the cross entropy loss takes into account both predictions ${\widehat{y}}_{1}^{i}$ and ${\widehat{y}}_{2}^{i}$ by averaging their combined values. The final loss function can be represented as follows:

16 $${{\text{L}}}^{{\text{i}}}=1/2[({{\text{L}}}_{{\text{i}}1}{\widehat{{\text{y}}}}_{1}^{{\text{i}}}+{{\text{L}}}_{{\text{i}}1}{\widehat{{\text{y}}}}_{2}^{{\text{i}}})+\mathrm{\alpha }({\text{KL}}\left({\widehat{{\text{y}}}}_{1}^{{\text{i}}}|\left|{\widehat{{\text{y}}}}_{2}^{{\text{i}}}\right)+{\text{KL}}\left({\widehat{{\text{y}}}}_{2}^{{\text{i}}}|\left|{\widehat{{\text{y}}}}_{1}^{{\text{i}}}\right)\right)\right]$$

where $\alpha$ is a learnable parameter. We consider both the prediction error and the difference between the model's output distributions. By optimizing the final loss function, we encourage the model to better fit the training data, thereby improving the model's generalization ability and robustness.

### Evaluation metrics

For the task of predicting drug combination synergy, the following metrics are used for evaluation: the area under the receiver operator characteristics curve (AUROC), the area under the precision − recall curve (AUPR), accuracy (ACC), balanced accuracy(BACC), precision (PREC), true positive rate (TPR), the Cohen’s Kappa value (KAPPA). ACC is used to describe the model's ability to distinguish between synergistic and antagonistic drug combinations. BACC and KAPPA are two metrics that consider the model's predictive ability for both synergistic and antagonistic drug combinations and are suitable for handling imbalanced datasets. TPR and TNR respectively represent the model's predictive accuracy for positive and negative samples. PREC measures the accuracy of the model in predicting drug pairs as synergistic combinations. Generally, the higher these metrics, the stronger the predictive ability of the model. The calculation formulas for these metrics are as follows:

17 $${\text{ACC}}=\frac{{\text{TP}}+{\text{TN}}}{{\text{TP}}+{\text{FP}}+{\text{TN}}+{\text{FN}}}$$

18 $${\text{TPR}}=\frac{{\text{TP}}}{{\text{TP}}+{\text{FN}}}$$

19 $${\text{TNR}}=\frac{{\text{TN}}}{{\text{FP}}+{\text{TN}}}$$

20 $${\text{BACC}}=\frac{{\text{TPR}}+{\text{TNR}}}{2}$$

21 $${\text{PREC}}=\frac{{\text{TP}}}{{\text{TP}}+{\text{FP}}}$$

22 $${\text{KAPPA}}=\frac{{{\text{p}}}_{{\text{o}}}-{{\text{p}}}_{{\text{e}}}}{1-{{\text{p}}}_{{\text{e}}}}$$

where $TP$, $FP$, $TN$, and $FN$ respectively represent the number of correctly identified synergistic drug combinations, the number of antagonistic drug combinations incorrectly identified as synergistic, the number of correctly identified antagonistic drug combinations, and the number of synergistic drug combinations incorrectly identified as antagonistic. ${p}_{o}$ is the ratio of the number of correctly classified samples to the total number of samples for each category, i.e., the overall classification accuracy. ${p}_{e}$ is the ratio of the sum of the products of the actual and predicted quantities for each category to the square of the total number of samples, representing the rate of chance agreement. These metrics evaluate the model's ability to accurately recognize different types of samples and the consistency of the labeling task, reflecting the overall performance of the model and helping us judge the reliability of the model in predicting the synergy of drug combinations.

### Experiment implementation

We use an RTX Nvidia 3090 GPU and is based on the PyTorch framework for training and testing. The Adam optimizer is used to update the model parameters. In the experiments, the batch size is set to 128; learning rate is set to 0.0001; dropout is set to 0.1; and cross-entropy is used as the loss function to measure the difference between the predicted results and the true labels.

## Results and discussion

### Performance comparison with other models

To evaluate the effectiveness of our model, it was compared with several existing methods on a benchmark dataset. These included methods based on machine learning for predicting drug combination synergy, such as Extreme Gradient Boosting(XGBoost), Random Forest(RF), Gradient Boosting Machines(GBM), Adaboost, Multilayer Perceptron(MLP), Support Vector Machines(SVM) and those based on deep learning, like DeepSynergy [23], TranSynergy [37], MGAE-DC [38], SDCNet [39], PRODeepSyn [40], DFFNDDS [41] and Deep Tensor Factorization(DTF) [42]. To delineate the distinctions between MFSynDCP and other deep learning-based approaches, we make the following summary for each deep learning model:

- *DeepSynergy:* DeepSynergy is a deep learning model that utilizes the chemical properties of two drugs and the gene expression of a cell line to forecast synergy scores. It utilizes a feedforward neural network to capture the potential pharmacological synergy between combinations of drugs.
- *TranSynergy:* TranSynergy integrates a Self-Attention Transformer to analyze drug synergy. It leverages input features such as drug-target interaction profiles, gene expression, and gene dependency profiles to compute a synergy score indicative of the effect of drug combinations on cell lines.
- *MGAE-DC:* The MGAE-DC framework utilizes a multi-channel graph autoencoder approach, with three distinct input channels designed to capture the effects of synergistic, additive, and antagonistic interactions among drugs. By employing a graph convolutional network, it acquires drug embeddings and applies an attention mechanism to amalgamate these embeddings across various cell lines, aiming to forecast drug synergies.
- *SDCNet:* SDCNet, a novel encoder-decoder architecture, is crafted to forecast cell line-specific Synergistic Drug Combinations (SDCs). It employs a Relational Graph Convolutional Network (R-GCN) for the acquisition and amalgamation of deep drug representations from various cell lines, utilizing an attention mechanism to refine the learning of features.
- *PRODeepSyn:* The PRODeepSy model integrates protein–protein interaction (PPI) networks with omics data via Graph Convolutional Networks to predict anticancer drug synergies.
- *DFFNDDS: *DFFNDDS is a deep learning model that predicts synergistic drug combinations by integrating a fine-tuned pretrained language model with a dual feature fusion mechanism, merging drug and cell line features at both bit-wise and vector-wise levels. This innovative approach ensures DFFNDDS establishes itself as a dependable tool for identifying effective drug combinations.
- *DTF:* DTF integrates a tensor-based framework with deep learning techniques to forecast the synergistic effects of drug pairs, primarily utilizing tensor factorization and a deep neural network for its predictions.

We divided the dataset into a training set and a test set, accounting for 90% and 10% of the data, respectively. Five-fold cross-validation was used in experiments on the training set, where training samples were randomly divided into five roughly equal subsets. Four subsets were used as the training dataset, and the remaining one served as a validation set for assessing the model's performance and tuning the hyperparameters. To further ensure the model's generalization ability and prevent overfitting, early stopping was applied during the training process. Specific results are shown in Table 1.

**Table 1:** Performance comparison of MFSynDCP and competitive methods on fivefold cross validation

Methods	AUROC	AUPR	ACC	BACC	PREC	TPR	KAPPA
Ours	$0.930\pm 0.005$	$0.929\pm 0.005$	$0.855\pm 0.006$	$0.855\pm 0.006$	$0.867\pm 0.012$	$0.863\pm 0.004$	$0.709\pm 0.012$
MGAE-DC	$0.922\pm 0.005$	$0.675\pm 0.010$	$0.851\pm 0.012$	$0.778\pm 0.005$	$0.723\pm 0.006$	$0.562\pm 0.003$	$0.603\pm 0.012$
SDCNet	$0.921\pm 0.007$	$0.920\pm 0.005$	$0.846\pm 0.007$	$0.832\pm 0.005$	$0.871\pm 0.005$	$0.795\pm 0.002$	$0.678\pm 0.008$
DFFNDDS	$0.912\pm 0.003$	$0.882\pm 0.005$	$0.821\pm 0.007$	$0.811\pm 0.003$	$0.821\pm 0.017$	$0.831\pm 0.013$	$0.661\pm 0.021$
XGBoost	$0.921\pm 0.005$	$0.922\pm 0.005$	$0.844\pm 0.005$	$0.844\pm 0.005$	$0.843\pm 0.007$	$0.840\pm 0.002$	$0.688\pm 0.009$
PRODeepDyn	$0.899\pm 0.005$	$0.922\pm 0.006$	$0.853\pm 0.007$	$0.853\pm 0.005$	$0.859\pm 0.007$	$0.856\pm 0.012$	$0.703\pm 0.003$
TranSynergy	$0.896\pm 0.007$	$0.892\pm 0.006$	$0.827\pm 0.013$	$0.827\pm 0.013$	$0.842\pm 0.006$	$0.801\pm 0.003$	$0.642\pm 0.013$
DTF	$0.892\pm 0.009$	$0.881\pm 0.008$	$0.814\pm 0.009$	$0.814\pm 0.009$	$0.822\pm 0.008$	$0.772\pm 0.031$	$0.633\pm 0.042$
DeepSynergy	$0.881\pm 0.005$	$0.874\pm 0.009$	$0.803\pm 0.007$	$0.803\pm 0.007$	$0.814\pm 0.011$	$0.752\pm 0.009$	$0.591\pm 0.048$
GBM	$0.852\pm 0.010$	$0.850\pm 0.007$	$0.772\pm 0.010$	$0.772\pm 0.010$	$0.773\pm 0.008$	$0.745\pm 0.014$	$0.544\pm 0.020$
Random Forest	$0.861\pm 0.010$	$0.850\pm 0.014$	$0.783\pm 0.012$	$0.783\pm 0.012$	$0.794\pm 0.016$	$0.751\pm 0.022$	$0.566\pm 0.024$
Adaboost	$0.828\pm 0.007$	$0.832\pm 0.010$	$0.743\pm 0.009$	$0.743\pm 0.009$	$0.746\pm 0.012$	$0.728\pm 0.006$	$0.486\pm 0.018$
MLP	$0.652\pm 0.024$	$0.640\pm 0.033$	$0.557\pm 0.045$	$0.560\pm 0.043$	$0.531\pm 0.042$	$0.924\pm 0.134$	$0.119\pm 0.085$
SVM	$0.586\pm 0.011$	$0.563\pm 0.011$	$0.542\pm 0.010$	$0.540\pm 0.010$	$0.534\pm 0.016$	$0.502\pm 0.067$	$0.081\pm 0.020$

Compared to other methods, our model achieved higher values in metrics such as AUROC, AUPR, ACC, BACC, TPR, KAPPA, indicating superior performance in the classification task of drug combination synergy prediction. Our model achieved an AUROC value of 0.930 ± 0.005, demonstrating a stronger ability to distinguish between synergistic and non-synergistic drug combinations. The AUPR value also reached 0.929 ± 0.005, indicating that the model maintains a high recall rate while achieving a high precision rate. In terms of the accuracy score (ACC), the model achieved a value of 0.855 ± 0.006, exhibiting higher accuracy compared to other methods. In terms of precision (PREC) and recall rate (TPR), the model reached a value of 0.867 ± 0.012, signifying its ability to correctly identify drug combinations with synergistic effects. To address the issue of class imbalance between synergistic and antagonistic drug combinations in the dataset, balanced accuracy (BACC) and KAPPA coefficient were used as evaluation metrics and reached values of 0.863 ± 0.004 and 0.709 ± 0.012, respectively. These performance metrics provided a comprehensive evaluation of various aspects of the model. Here, it is noteworthy that MGAE-DC, SDCNet and the classical machine learning methods, such as XGB, also obtain competitive performance, but nevertheless still inferior to our method. Overall, our method achieves superior performance on most evaluation metrics compared to the advanced deep learning methods and classical machine learning methods.

To further confirm the statistical significance of the superiority of our model, we conducted t-tests for a statistical analysis on three important metrics: AUROC, AUPR, and ACC, comparing the performance differences between our model and other benchmark models. As shown in Table 2, the obtained p-values consistently fall below the standard significance level of 0.05, indicating that our model significantly outperforms all compared models in a statistical sense. These results further showcase the exceptional performance of our model, MFSynDCP, on the aforementioned performance metrics, validating its effectiveness in predicting synergistic cancer drug combinations.

**Table 2:** P-value comparison of MFSynDCP and comparative methods using t-test

Methods	p-value t-test of AUROC	p-value t-test of AUPR	p-value t-test of ACC
MGAE-DC	$7.28\times {10}^{-3}$	$7.73\times {10}^{-27}$	$4.72\times {10}^{-3}$
SDCNet	$4.42\times {10}^{-2}$	$5.13\times {10}^{-3}$	$3.56\times {10}^{-5}$
DFFNDDS	$2.13\times {10}^{-6}$	$1.01\times {10}^{-11}$	$5.03\times {10}^{-13}$
XGBoost	$1.36\times {10}^{-3}$	$4.87\times {10}^{-4}$	$1.22\times {10}^{-5}$
PRODeepDyn	$7.22\times {10}^{-9}$	$3.73\times {10}^{-3}$	$6.71\times {10}^{-3}$
TranSynergy	$9.63\times {10}^{-9}$	$7.67\times {10}^{-12}$	$1.15\times {10}^{-3}$
DTF	$8.72\times {10}^{-10}$	$3.25\times {10}^{-12}$	$1.71\times {10}^{-10}$
DeepSynergy	$5.34\times {10}^{-13}$	$4.69\times {10}^{-13}$	$3.28\times {10}^{-15}$
GBM	$3.19\times {10}^{-13}$	$7.21\times {10}^{-16}$	$7.98\times {10}^{-16}$
Random Forest	$6.01\times {10}^{-13}$	$3.54\times {10}^{-10}$	$1.66\times {10}^{-13}$
Adaboost	$3.37\times {10}^{-14}$	$9.82\times {10}^{-16}$	$5.16\times {10}^{-17}$
MLP	$9.69\times {10}^{-18}$	$8.65\times {10}^{-20}$	$1.77\times {10}^{-13}$
SVM	$6.76\times {10}^{-24}$	$1.49\times {10}^{-24}$	$1.52\times {10}^{-25}$

In the experiments, selections were made for hyperparameter values, including the learning rate, dimension of the GAT layer, dropout ratio, and batch size. Notably, when the learning rate was set to 0.001, the dimension of the GAT layer to 64, the dropout ratio to 0.1, and the batch size to 128, the model was more effective in extracting drug features. This combination of parameters not only improved the model's performance on the training set but also demonstrated good generalization ability on the validation set. Further, it was found that fine-tuning the dimension of the GAT layer significantly impacts the model's sensitivity in handling complex drug molecular structures. An appropriate dropout ratio helps prevent overfitting, ensuring the stability of the model's training.

### Evaluation on independent test dataset

**Fig. 4:**
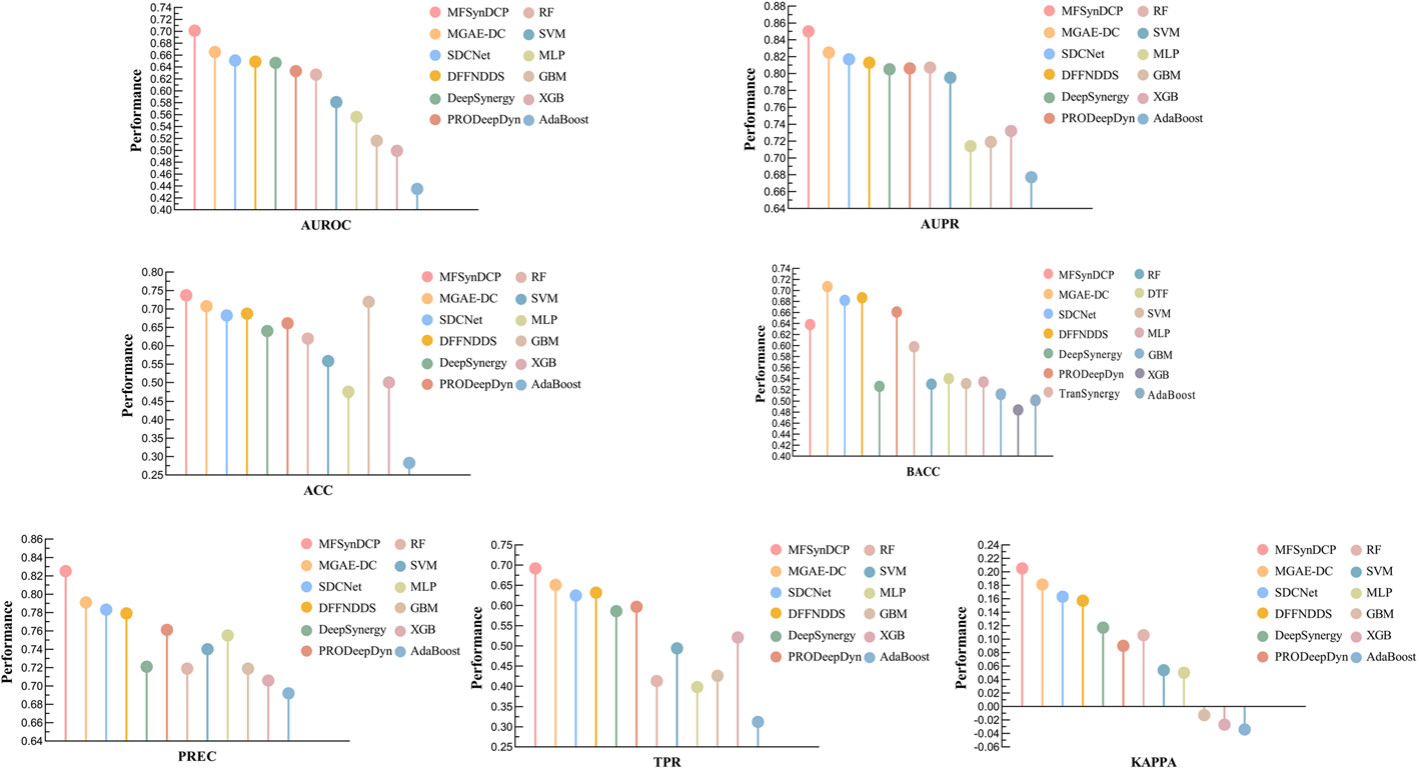
Performance of MFSynDCP and its variants on the independent test dataset released by AstraZeneca

To further validate the generalization ability of our model on new datasets, the study also employed a large drug combination dataset released by AstraZeneca [43] in 2019 as an independent test set to evaluate the performance of MFSynDCP and other benchmark methods. This dataset is the result of the AstraZeneca-Sanger Drug Combination Prediction DREAM Challenge, a collaboration between AstraZeneca and the Sanger Institute, aimed at exploring fundamental characteristics of effective combination therapy and synergistic drug behavior. The dataset consists of 668 novel drug-drug-cell line triplets, comprising 57 drugs and 24 cell lines.By training on the benchmark dataset and testing on this independent test set, as shown in Fig. 4, our model demonstrated favorable performance, correctly predicting 492 drug combination pairs. Moreover, our model outperformed other comparison methods across all evaluation metrics.

**Fig. 5:**
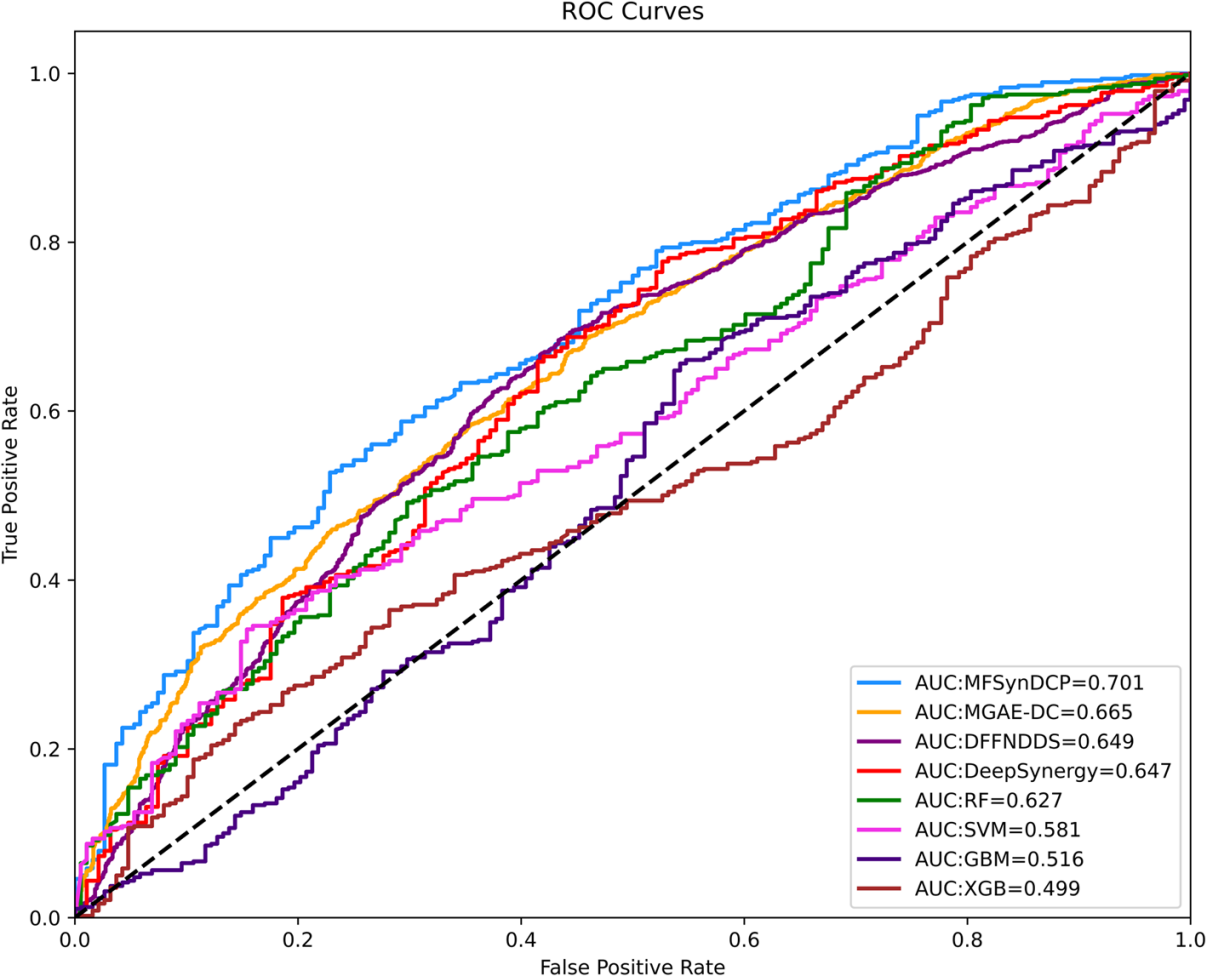
ROC curves of MFSynDCP and competitive methods on independent test dataset

To more intuitively demonstrate the effectiveness of our model, we selected three deep learning approaches and four machine learning methods for the plotting of ROC curves, as shown in Fig. 5. The graphical representation clearly illustrates that our model achieved a significant AUROC score of $0.701\pm 0.12$, surpassing other competing models. In contrast, the ROC curves of some benchmark models closely resembled random predictions, indicating their limited predictive accuracy for drug combinations. However, our model consistently exhibited exceptional performance on the test set of this challenge, thereby further validating its excellent generalization capability and practical applicability.

### Ablation study

**Fig. 6:**
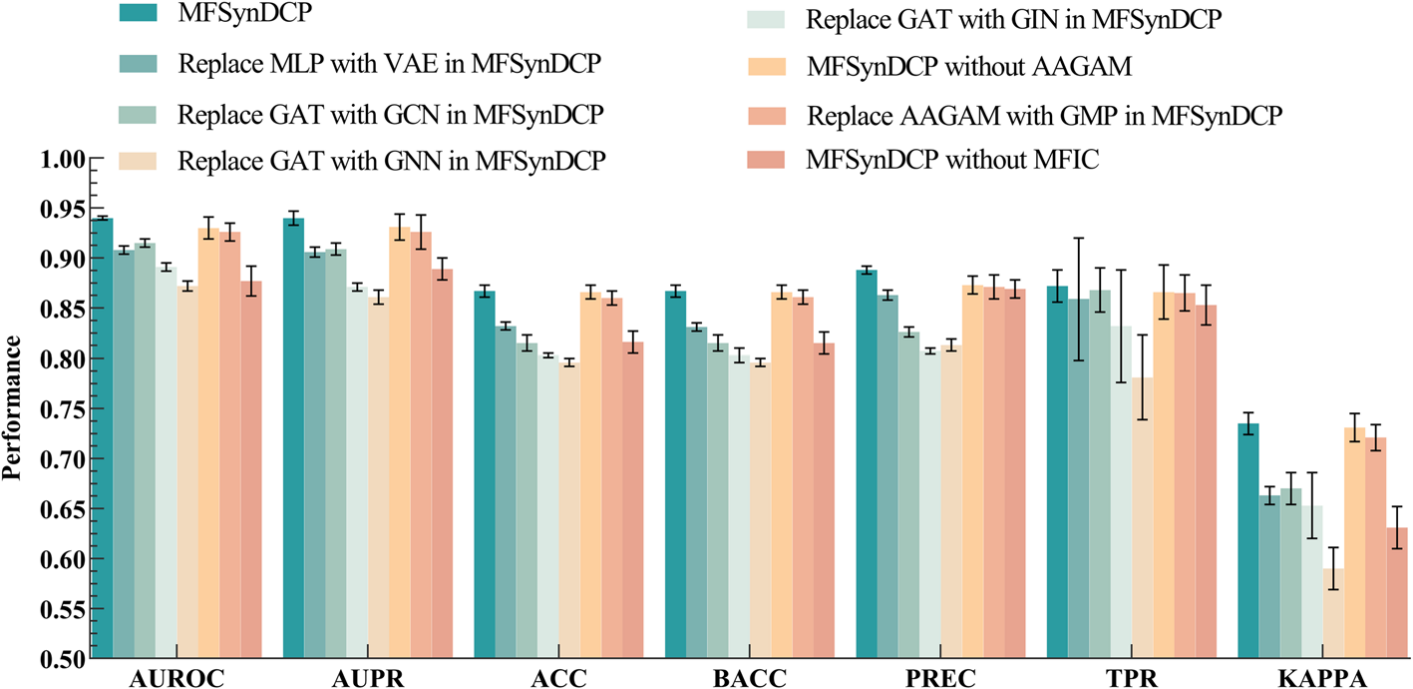
The performance of our proposed MFSynDCP and its variants

To investigate the importance and contribution of each component in the model, we conducted an ablation analysis by removing or replacing some model components. Specifically, we compared the results of MFSynDCP under the following conditions: (i) the proposed MFSynDCP, (ii) replace MLP with Variational Autoencoder (VAE) in MFSynDCP, (iii) replace GAT with GCN in MFSynDCP, (iv) replace GAT with Graph Isomorphism Network (GIN) in MFSynDCP, (v) replace GAT with GNN in MFSynDCP (vi) MFSynDCP without AAGAM, (vii) replace AAGAM with the global mean pooling (GMP) in MFSynDCP, (viii) MFSynDCP without MFIC. We conducted a fivefold cross-validation test based on the training dataset for comparison. The results on the benchmark dataset are summarized in the figure below. Figure 6 shows the experimental results of our model MFSynDCP compared with the other four variants.

The results indicate that the complete MFSynDCP framework achieved the best predictive performance in the 7 evaluation metrics, demonstrating its effectiveness. Specifically, we observed that GAT outperforms GCN, GIN, and GNN in extracting key chemical features. Through the adaptive attention mechanism of GAT, it can better capture important features and interaction information among drugs, thereby improving the predictive performance of the model. In contrast, GCN, GIN, and GNN exhibit limitations in utilizing the complex relationships among drugs in certain aspects.

As can be observed in Fig. 6, in terms of encoding the genomic features of cancer cells, the use of MLP outperforms VAE. A possible reason for this could be the flexible network architecture and feature learning capability of MLP, which allows it to capture the key nonlinear relationships and complex features within the cancer cell genome more effectively than VAE.

Notably, the complete framework scored lower on the TPR metric compared to the model without the AAGAM, which might be due to the class imbalance between synergistic and antagonistic drug combinations in the dataset. To address this issue, BACC and KAPPA coefficients can be used for assessment, where the complete model achieved the highest performance scores.

The experimental results prove that the AAGAM in our model performs better than the versions without adaptive attention mechanisms and those using global mean pooling for graph aggregation. This is likely because, for molecular graphs, the global mean pooling method treats every substructure as equally important and simply averages the embeddings of all nodes. In contrast, our proposed AAGAM utilizes the interaction information of drug pairs, not just the molecular graph of a single drug, to obtain attention scores for each substructure, thereby achieving better performance compared to the other two comparison models.

Furthermore, it can be concluded that the models without the AAGAM and those replacing AAGAM with GMP did not show a significant difference in the AUROC, AUPR, ACC, BACC, TPR, and KAPPA metrics, and their performance was lower than the predictive indicators of our proposed model. This underscores the important role of our proposed adaptive attention mechanism in the graph aggregation module within this model. Simultaneously, the MFIC design makes a greater contribution to learning drug features compared to the AAGAM proposed in our study, possibly due to its effective handling and integration of feature information from different sources, including both drugs and cell lines. The MFIC led fusion module plays a key role in ensuring high-quality predictions of drug synergistic effects. The experimental results according to AUROC, AUPR, ACC, BACC, TPR, and KAPPA indicate that the absence of MFIC led to a significant drop in model performance, suggesting that the MFIC-led fusion module effectively captures the synergistic effects between features. The higher scores in the PREC metric could also confirm the imbalance in the dataset; if there are fewer positive samples and more negative samples, the model may be biased towards predicting samples as negative to achieve a higher accuracy rate. Moreover, we observed that the complete MFSynDCP framework outperformed the other ablation scenarios in six metrics, further validating the importance of each component.

In summary, the experimental results clearly demonstrate that the adaptive attention mechanism and the MFIC-led fusion module play a crucial role in enhancing model performance. Their combination is capable of more comprehensively capturing the features of drug synergistic effects.

### The impact of the input sequence of drug combination data on predictive performance

**Fig. 7:**
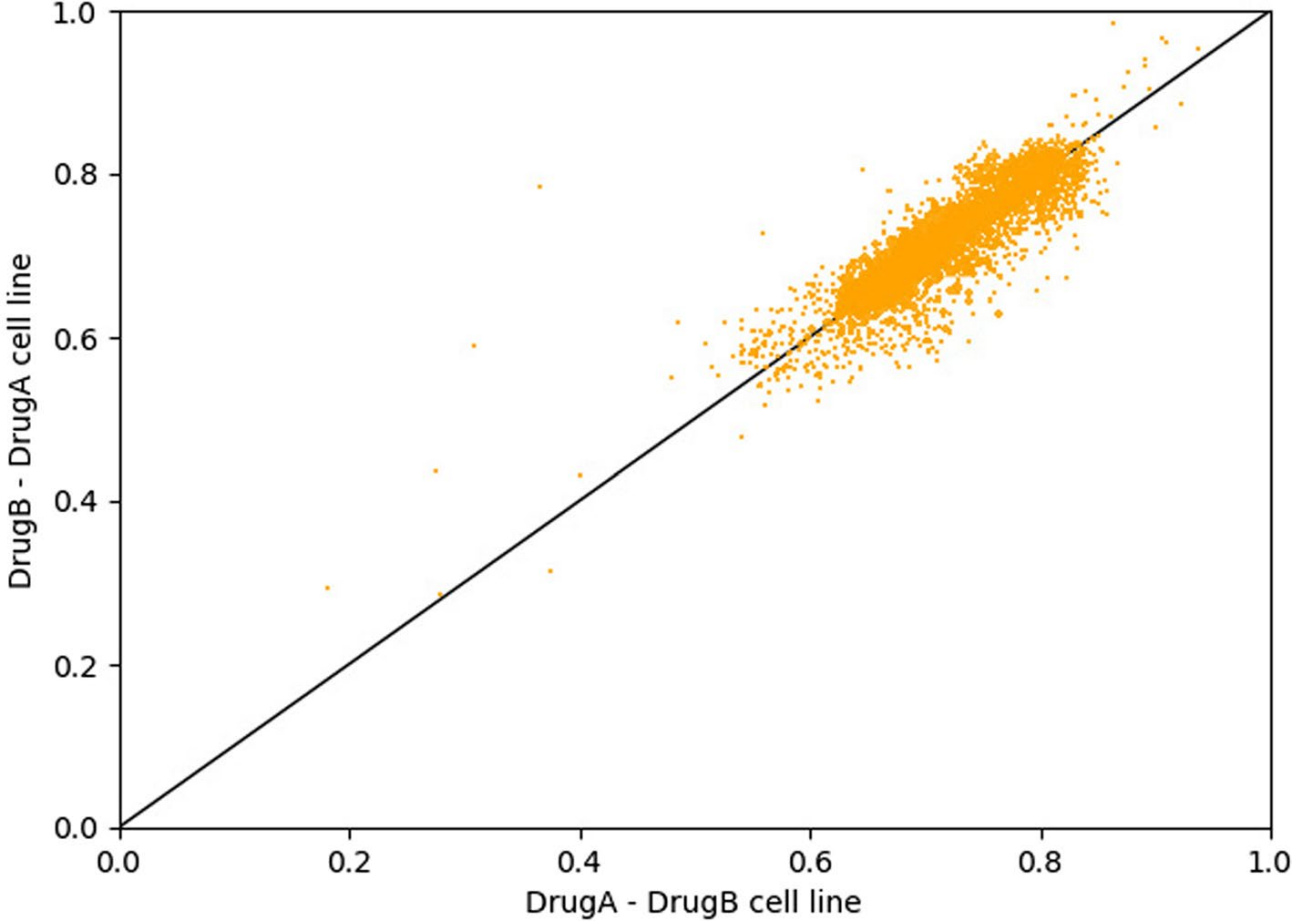
Scatter plot of collaborative scores obtained based on different input orders of two drugs

To mitigate the impact of drug order on the model's prediction results, during the training process, we treated [drug A, drug B, cell line] samples and [drug B, drug A, cell line] samples as two distinct input samples. This approach allowed us to examine the effect of different input feature orders on predicting synergy scores. As shown in Fig. 7, we observed that the prediction results under different input feature orders are concentrated near the diagonal line, with a Pearson correlation coefficient reaching 0.9. This indicates that our model is not sensitive to the order of drug combinations; accurate predictions are generated regardless of whether it's drug A-drug B or drug B-drug A. This further verifies the robustness and reliability of our model. Additionally, we observed that both the ROC AUC (Area Under the Receiver Operating Characteristic Curve) and PR AUC (Precision-Recall Area Under Curve) for [drug A, drug B] and [drug B, drug A] reached or were close to 0.93, further proving the excellent performance of our model in this study.

### The revelation of crucial chemical substructures in drugs

Deep learning models are often viewed as black boxes, and their lack of interpretability limits their further application in many fields, especially in practical scenarios of computational-aided drug discovery. To address this issue and explore the key substructures in drug combination prediction, we utilized the attention mechanism to visualize the critical substructures of drug pairs.

The MFSynDCP model proposed in this study employs a message-passing mechanism between nodes to update each node's information. This allows each node to capture information from its neighboring nodes and gradually accumulate and integrate information from surrounding nodes, enriching its feature representation. In this model, each neuron in the GAT network is connected to neighboring nodes from the previous layer through a set of learnable weights, enabling the neuron to acquire information from its neighbors and incorporate it into its feature expression. Furthermore, we introduced an adaptive attention mechanism-based graph aggregation module. This module assigns attention scores to each substructure of a drug and performs a weighted summation of all nodes' embedding vectors, resulting in a graph-aggregated representation of the drug. This process reveals the key chemical substructures that play a crucial role in synergy prediction. Therefore, the final drug feature representation actually contains information about the surrounding chemical substructures, including valency, solubility, and other physicochemical properties. This inspired our exploration of the attention mechanism in revealing important chemical substructures.

**Fig. 8:**
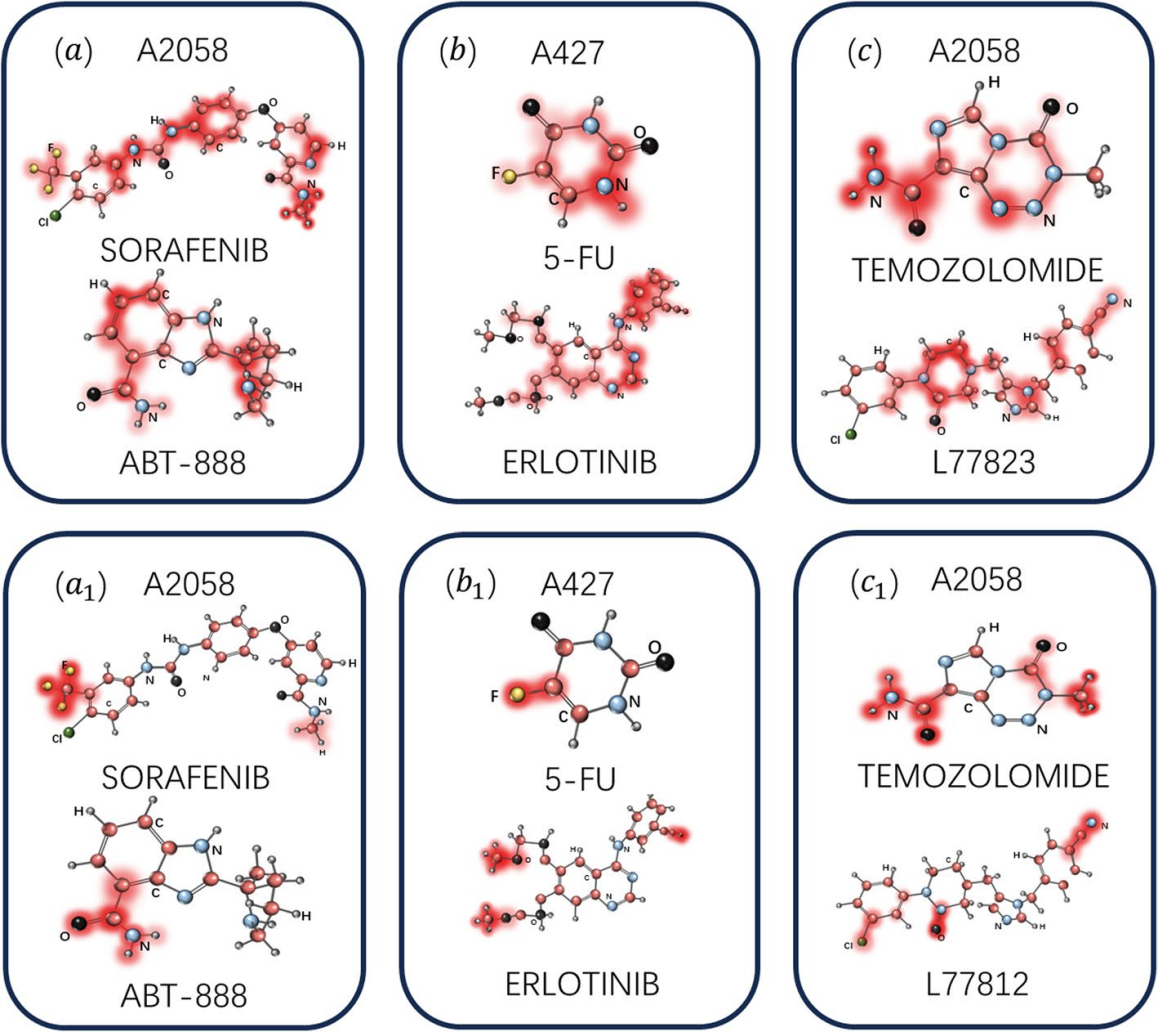
The visualization results for three randomly selected drug pairs are presented. Figure 8a-8c display the visualization of attention scores for these three drug pairs before training. Figure 8${a}_{1}$-${c}_{1}$ shows the visualization of attention scores for these three drug pairs during the model training process, where deeper colors indicate more important substructures

Specifically, the attention scores calculated using formulas 4 and 5 are used to represent the importance levels of corresponding substructures. These substructures' importance is visualized using different colors. Figure 8 displays the visualization results for three randomly selected drug pairs (ABT-888 and SORAFENIB, 5-FU and Erlotinib, L778123 and TEMOZOLOMIDE). In the initial stages of training, the attention scores show a more uniform distribution, indicating that the model has not yet focused on key structures with significant influence. However, as training progresses, the model gradually starts to assign higher importance to certain specific structures compared to others. Fig. 8*a*_1_–*c*_1_ presents the visualization results obtained after the model's training is complete, where deeper colors reflect more important substructures.

Taking Fig. 8b and ${b}_{1}$ as examples for explanation: A427 [44] is a human non-small cell lung cancer cell line, while 5-FU [45] and Erlotinib [46] are two drugs commonly used in lung cancer treatment. These drugs can be used in combination therapy. 5-FU and Erlotinib have been shown to have a more effective growth inhibitory effect on the A427 cell line. Our model successfully identified the amide group as an important chemical structure, which plays a key role in biomolecules, including many clinically approved drugs. Amides are widely present in drugs, not only because of their stability but also because their polarity allows drugs containing amide groups to interact with biological receptors and enzymes. This result demonstrates the good interpretability of our model.

### The prediction of new synergistic drug combinations

The pursuit of innovative and effective drug combinations remains a cornerstone in the fight against cancer, presenting a complex yet crucial challenge in medical research. We introduce a refined methodological approach aimed at identifying synergistic drug combinations, effectively capitalizing on the sophisticated capabilities of the MFSynDCP model. Our approach integrates computational modeling with clinical predictive analysis, aiming to identify novel drug combinations that have the potential to alter current treatment modalities, thereby offering new research pathways and therapeutic strategies in the field of cancer treatment.

To assess the model's potential in discovering new synergistic drug combinations, we trained our MFSynDCP model using the O'Neil drug combination dataset. To generate candidate drug combinations, we selected 25 small-molecule anticancer chemical drugs approved by the U.S. Food and Drug Administration (FDA), removing drug combinations that duplicated those in the benchmark dataset. We then used our MFSynDCP model to predict the synergy of the final candidate drug pairs. Extensive literature searches were conducted to validate whether the model could identify new synergistic drug combinations.

In this study, new drug combinations were predicted using the widely studied A375 cancer cell line [47], forecasting unknown [drug, drug, cell line] triplets. We focused particularly on the top 7 ranked untested triplets in the prediction scores and conducted a non-exhaustive literature search. We found that 3 of the predicted drug combinations were consistent with previous research or clinical trial observations. For instance, the combination of Erlotinib and Regorafenib was used for the treatment of hepatocellular carcinoma, successfully overcoming the interference of epidermal growth factor [48]. These examples illustrate that MFSynDCP can successfully predict drug combinations consistent with previous research or clinical trial observations, further validating its potential in discovering new synergistic drug combinations.

**Fig. 9:**
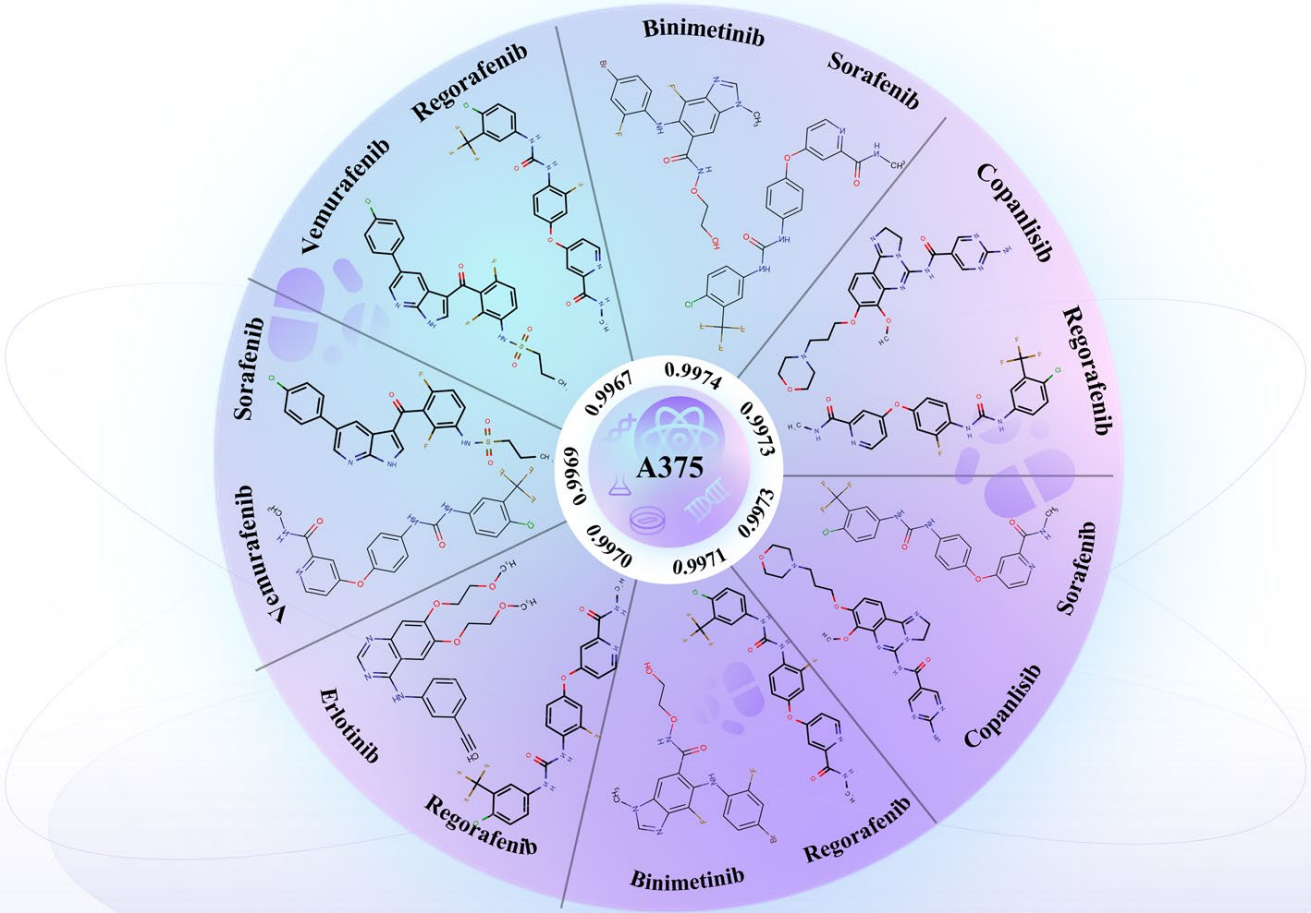
The top 7 novel synergistic combinations predicted on A375 cancer cell line

The predicted new synergistic drug combinations each consist of two drugs, and each combination is assigned a predictive score reflecting its potential synergistic efficacy. Additionally, the predictive scores for all listed drug combinations are exceptionally high (close to 1), indicating these combinations show substantial potential for synergy in the model. At least three of these predicted drug combinations are consistent with existing research or clinical trial observations, enhancing the reliability of the predictions. All these predictions are made for the A375 cancer cell line, a melanoma (skin cancer) cell line. This specificity suggests that these combinations may not be equally effective against other types of cancer cells. Future research could focus on validating the actual efficacy and potential synergistic mechanisms of those combinations that are supported by literature but have not yet entered clinical trial stages, as well as those completely unsupported by existing literature. Overall, Fig. 9 demonstrates the capability of the MFSynDCP model in predicting potentially effective new synergistic drug combinations. It offers a promising beginning for further experimental and clinical research, highlighting the model's utility in guiding hypothesis generation and decision-making in drug development and personalized medicine.

## Conclusions

In this paper, we proposed a deep graph neural network model named MFSynDCP, which is guided by a multi-source feature interactive learning controller and employs an adaptive attention mechanism for predicting the synergy of anticancer drug combinations. Specifically, the SMILES features of drugs are first transformed into drug molecular structure graphs, and a Graph Attention Network (GAT) is used to extract structural information from drug pairs. An adaptive attention mechanism-based graph aggregation module was designed to unearth the most critical chemical substructures for synergy prediction. Additionally, an innovative multi-source feature interactive learning controller was constructed to enhance the representation of drug pairs, enabling the fusion of multi-source data from drugs and cell lines and learning the interaction information between them. We also explored the learning process of MFSynDCP, uncovering the mechanisms of drug synergy among substructures, which provided a level of interpretability to the model and supported the explanation of drug synergy mechanisms. Our performance comparison experiments demonstrated that MFSynDCP outperforms other competitive methods.

However, the MFSynDCP model has certain limitations. The study focused solely on features of drugs and cell lines for synergy prediction, without considering the potential of biomedical knowledge graph methods in predicting effective combinations for diseases. In the future, we plan to integrate biomedical knowledge graphs to further enhance the overall performance of predicting synergistic anticancer drug combinations. Additionally, we recognize the importance of exploring gene contributions in synergy prediction and plan to incorporate this aspect into our future research endeavors. This will allow us to gain a more comprehensive understanding of the factors influencing synergy prediction and improve the predictive capabilities of our model.

## Data Availability

All data generated or analyzed during this study are included in this article.

## Code availability

The source code is available from GitHub at https://github.com/kkioplkg/MFSynDCP.

## References

[1] Humphrey RW, Brockway-Lunardi LM, Bonk DT, Dohoney KM, Doroshow JH, Meech SJ (2011). Opportunities and challenges in the development of experimental drug combinations for cancer. J Natl Cancer Inst.

[2] Wu L, Wen Y, Leng D, Zhang Q, Dai C, Wang Z (2022). Machine learning methods, databases and tools for drug combination prediction. Brief Bioinform.

[3] Jaaks P, Coker EA, Vis DJ, Edwards O, Carpenter EF, Leto SM (2022). Effective drug combinations in breast, colon and pancreatic cancer cells. Nature.

[4] Yu J, Mu Q, Fung M, Xu X, Zhu L, Ho RJJP (2022). Challenges and opportunities in metastatic breast cancer treatments: nano-drug combinations delivered preferentially to metastatic cells may enhance therapeutic response. Pharmacol Ther.

[5] Zhu C, Guan X, Zhang X, Luan X, Song Z, Cheng X (2022). Targeting KRAS mutant cancers: from druggable therapy to drug resistance. Mol Cancer.

[6] Yardley DA (2013). Drug resistance and the role of combination chemotherapy in improving patient outcomes. Int J Breast Cancer.

[7] Lin R, Yin G (2017). Bayesian optimal interval design for dose finding in drug-combination trials. Stat Methods Med Res.

[8] Lin GL, Wilson KM, Ceribelli M, Stanton BZ, Woo PJ, Kreimer S (2019). Therapeutic strategies for diffuse midline glioma from high-throughput combination drug screening. Sci Transl Med.

[9] Kim H, Lee SJ, Lee IK, Min SC, Sung HH, Jeong BC (2020). Synergistic effects of combination therapy with AKT and mTOR inhibitors on bladder cancer cells. Int J Mol Sci.

[10] Costa A, Campos D, Reis C, Gomes C (2020). Targeting glycosylation: a new road for cancer drug discovery. Trends Cancer.

[11] Lu Y, Chan Y-T, Tan H-Y, Li S, Wang N, Feng Y (2020). Epigenetic regulation in human cancer: the potential role of epi-drug in cancer therapy. Mol Cancer.

[12] Zhao X-M, Iskar M, Zeller G, Kuhn M, Van Noort V, Bork P (2011). Prediction of drug combinations by integrating molecular and pharmacological data. PLoS Comput Biol.

[13] Beam AL, Drazen JM, Kohane IS, Leong T-Y, Manrai AK, Rubin EJ (2023). Artificial intelligence in medicine. N Engl J Med.

[14] De Ville B (2013). Decision trees. Wiley Interdiscip Rev Comput Stat.

[15] Breiman L (2001). Random forests. Mach Learn.

[16] Noble WS (2006). What is a support vector machine?. Nat Biotechnol.

[17] Šícho M, Luukkonen S, van den Maagdenberg HW, Schoenmaker L, Béquignon OJ, van Westen GJ (2023). DrugEx: deep learning models and tools for exploration of drug-like chemical space. J Chem Inf Model.

[18] Mei S (2022). A machine learning framework for predicting synergistic and antagonistic drug combinatorial efficacy. J Math Chem.

[19] Janizek JD, Dincer AB, Celik S, Chen H, Chen W, Naxerova K (2023). Uncovering expression signatures of synergistic drug responses via ensembles of explainable machine-learning models. Nat Biomed Eng.

[20] Julkunen H, Cichonska A, Gautam P, Szedmak S, Douat J, Pahikkala T (2020). Leveraging multi-way interactions for systematic prediction of pre-clinical drug combination effects. Nat Commun.

[21] Hall MA (1999). Correlation-based feature selection for machine learning.

[22] Shaheen F, Verma B, Asafuddoula M. Impact of automatic feature extraction in deep learning architecture. In: 2016 International conference on digital image computing: techniques and applications (DICTA). IEEE; 2016. pp. 1–8. 10.1109/DICTA.2016.7797053

[23] Preuer K, Lewis RP, Hochreiter S, Bender A, Bulusu KC, Klambauer GJB (2018). DeepSynergy: predicting anti-cancer drug synergy with Deep Learning. Bioinformatics.

[24] Rafiei F, Zeraati H, Abbasi K, Ghasemi JB, Parsaeian M, Masoudi-Nejad AJB (2023). DeepTraSynergy: drug combinations using multimodal deep learning with transformers. Bioinformatics.

[25] Yang J, Xu Z, Wu WK, Chu Q, Zhang Q (2021). GraphSynergy: a network-inspired deep learning model for anticancer drug combination prediction. J Am Med Inform Assoc.

[26] O'Neil J, Benita Y, Feldman I, Chenard M, Roberts B, Liu Y (2016). An unbiased oncology compound screen to identify novel combination strategies. Mol Cancer Ther.

[27] Di Veroli GY, Fornari C, Wang D, Mollard S, Bramhall JL, Richards FM (2016). Combenefit: an interactive platform for the analysis and visualization of drug combinations. Bioinformatics.

[28] Goldoni M, Johansson C (2007). A mathematical approach to study combined effects of toxicants in vitro: evaluation of the bliss independence criterion and the Loewe additivity model. Toxicol In Vitro.

[29] Weininger D (1988). SMILES, a chemical language and information system. 1. Introduction to methodology and encoding rules. J Chem Inf Comput Sci.

[30] Wishart DS, Feunang YD, Guo AC, Lo EJ, Marcu A, Grant JR (2018). DrugBank 5.0: a major update to the DrugBank database for 2018. Nucleic Acids Res.

[31] Bento AP, Hersey A, Félix E, Landrum G, Gaulton A, Atkinson F (2020). An open source chemical structure curation pipeline using RDKit. J Cheminformatics.

[32] Barretina J, Caponigro G, Stransky N, Venkatesan K, Margolin AA, Kim S (2012). The cancer cell line encyclopedia enables predictive modelling of anticancer drug sensitivity. Nature.

[33] Velculescu VE, Madden SL, Zhang L, Lash AE, Yu J, Rago C (1999). Analysis of human transcriptomes. Nat Genet.

[34] Ramsundar B (2018). Molecular machine learning with DeepChem.

[35] Yang W, Soares J, Greninger P, Edelman EJ, Lightfoot H, Forbes S (2012). Genomics of drug sensitivity in cancer (GDSC): a resource for therapeutic biomarker discovery in cancer cells. Nucleic Acids Res.

[36] Derrien T, Johnson R, Bussotti G, Tanzer A, Djebali S, Tilgner H (2012). The GENCODE v7 catalog of human long noncoding RNAs: analysis of their gene structure, evolution, and expression. Genome Res.

[37] Liu Q, Xie L (2021). TranSynergy: mechanism-driven interpretable deep neural network for the synergistic prediction and pathway deconvolution of drug combinations. PLoS Comput Biol.

[38] Zhang P, Tu S (2023). MGAE-DC: predicting the synergistic effects of drug combinations through multi-channel graph autoencoders. PLoS Comput Biol.

[39] Zhang P, Tu S, Zhang W, Xu L (2022). Predicting cell line-specific synergistic drug combinations through a relational graph convolutional network with attention mechanism. Brief Bioinform.

[40] Wang X, Zhu H, Jiang Y, Li Y, Tang C, Chen X (2022). PRODeepSyn: predicting anticancer synergistic drug combinations by embedding cell lines with protein–protein interaction network. Brief Bioinform.

[41] Xu M, Zhao X, Wang J, Feng W, Wen N, Wang C (2023). DFFNDDS: prediction of synergistic drug combinations with dual feature fusion networks. J Cheminformatics.

[42] Sun Z, Huang S, Jiang P, Hu P (2020). DTF: deep tensor factorization for predicting anticancer drug synergy. Bioinformatics.

[43] Menden MP, Wang D, Mason MJ, Szalai B, Bulusu KC, Guan Y (2019). Community assessment to advance computational prediction of cancer drug combinations in a pharmacogenomic screen. Nat Commun.

[44] Parry JJ, Eiblmaier M, Andrews R, Meyer LA, Higashikubo R, Anderson CJ (2007). Characterization of somatostatin receptor subtype 2 expression in stably transfected A-427 human cancer cells. Mol Imaging.

[45] Wigmore PM, Mustafa S, El-Beltagy M, Lyons L, Umka J, Bennett G, Raffa RB, Tallarida RJ (2010). Effects of 5-FU. Chemo fog.

[46] Bareschino MA, Schettino C, Troiani T, Martinelli E, Morgillo F, Ciardiello F (2007). Erlotinib in cancer treatment. Ann Oncol.

[47] Zhang YP, Li YQ, Lv YT, Wang JM (2015). Effect of curcumin on the proliferation, apoptosis, migration, and invasion of human melanoma A375 cells. Genet Mol Res.

[48] D’Alessandro R, Refolo MG, Lippolis C, Carella N, Messa C, Cavallini A (2015). Modulation of Regorafenib effects on HCC cell lines by epidermal growth factor. Cancer Chemother Pharmacol.

